# Complete Genome Sequence of *Haemophilus influenzae* Strain 375 from the Middle Ear of a Pediatric Patient with Otitis Media

**DOI:** 10.1128/genomeA.01245-14

**Published:** 2014-12-04

**Authors:** Joshua Chang Mell, Sunita Sinha, Sergey Balashov, Cristina Viadas, Christopher J. Grassa, Garth D. Ehrlich, Corey Nislow, Rosemary J. Redfield, Junkal Garmendia

**Affiliations:** a Centers for Genomic Sciences and Advanced Microbial Processing, Institute for Molecular Medicine & Infectious Disease, Department of Microbiology and Immunology, Drexel University College of Medicine, Philadelphia, Pennsylvania, USA; b Biodiversity Research Centre, University of British Columbia, Vancouver, BC, Canada; c Department of Zoology, University of British Columbia, Vancouver, BC, Canada; d Department of Pharmaceutical Sciences, University of British Columbia, Vancouver, BC, Canada; e Genomic Core Facility, Institute for Clinical and Translational Research, Drexel University College of Medicine, Philadelphia, Pennsylvania, USA; f Instituto de Agrobiotecnología, CSIC-Universidad Pública Navarra-Gobierno, Navarra, Spain; g CIBERES, Madrid, Spain; h Department of Botany, University of British Columbia, Vancouver, BC, Canada

## Abstract

Originally isolated from a pediatric patient with otitis media, *Haemophilus influenzae* strain 375 (Hi375) has been extensively studied as a model system for intracellular invasion of airway epithelial cells and other pathogenesis traits. Here, we report its complete genome sequence and methylome.

## GENOME ANNOUNCEMENT

*Haemophilus influenzae* is a diverse bacterium, usually associated with human nasopharyngeal carriage but it can also be a potent pathogen. Although an effective vaccine against meningitis-causing type b strains is in wide use, nontypeable *H. influenzae* (NTHi) remains a common problem in patients with chronic respiratory conditions and pediatric ear infections (1, 2). The NTHi otitis media isolate Hi375 has been extensively used in studies of bacterial pathogenesis, particularly with respect to intracellular invasion of airway epithelia, outer membrane physiology, and animal models of pathogenesis (3–7).

Genomic DNA was extracted by the CTAB method (8), and sequencing libraries were constructed according to the manufacturers’ instructions using Nextera XT for Illumina and the 6-kb insert protocol for PacBio. Illumina sequencing was part of multiplexed HiSeq RapidRuns, and ~3.8 × 10^7^ read pairs (2 × 101 nt) were collected for Hi375 (~4,000-fold coverage). PacBio sequencing (v 2.1.0) was performed using a single SMRTcell with P4-C2 chemistry. A 2-h movie generated 44,007 polymerase reads (*N*_50_ = 5,022 nucleotide (nt); postfiltered subreads, *N*_50_ = 3,116 nt).

*De novo* assembly of Illumina reads trimmed adapters with Trimmomatic (9), merged overlapping reads with COPE (10), and assembled with RAY (11), as previously described (12), yielding 21 contigs. This assembly was reconciled using CISA (13) with another partial assembly of Hi375 (14), producing a merged assembly of 16 contigs covering 1,824,471 bp.

*De novo* assembly of PacBio data with the HGAP assembler (15) (v3beta) yielded a single contig with mean coverage of 66-fold (1 <3-kb contig with coverage <10-fold was discarded). Circular closure used Minimus2 (http://amos.sourceforge.net/wiki/index.php/Minimus2) to trim the ends and permute the genome to begin at the DnaA gene (identified by BLAST), followed by Quiver-based error correction (15) for a final closed genome size of 1,850,897 bp. Assembly accuracy was verified using Mauve (16) to reorder Illumina contigs against the complete assembly, finding perfect synteny. Illumina read pairs were aligned to the complete assembly using bwa mem (17) and sambamba (https://github.com/lomereiter/sambamba). Subsequently samtools mpileup and bcftools view (18) identified no variants with quality of >30.

The Pacific Biosciences “Modification and Motif Analysis” pipeline (v1) identified six 6-methyladenine motifs (bold underlined positions at Ts indicating methylation on the reverse complement): GATC, CCGA**A**, G**A**CCN _6_GTT, **A**TGN _6_CC**T**, TC**A**N _6_**T**RCC, A**A**CN _6_R**T**C. Additionally, an unknown cytosine modification motif was identified (G**C**GCGCBHV).

Results with a streptomycin-resistant (Str^r^) derivative—created by transformation with a PCR fragment from a multidrug resistant Rd derivative, MAP7 (coordinates 599,059 to 602,433 of the Rd genome, NC_000907.1) were comparable to those described above for Hi375. A single circularized contig was generated, and short reads agreed with the assembly. Eight single-nucleotide variants distinguished this strain from Hi375. As expected, all were clustered at *rpsL* (30S ribosomal protein S12), including the Str^r^ allele, an A → G transition at position 444,369. The remaining variants were the next seven that distinguish Hi375 from Rd.

Annotation by the NCBI prokaryotic genome annotation pipeline found the Hi375 chromosome contains 1,699 coding sequences, 6 rRNA clusters, and 59 tRNAs, covering all 20 amino acids including selenocysteine. We expect this complete genome to facilitate molecular genomics investigations into NTHi pathogenesis.

### Nucleotide sequence accession number.

The complete genome of nontypeable *Haemophilus influenzae* strain 375 was submitted to NCBI under the accession number CP009610. This is the first version of the complete sequence.
